# Supplementary material for: TAPB: an interventional debiasing framework for alleviating target prior bias in drug-target interaction prediction
Source: Nat Commun. 2025 Dec 2;16:10867. doi: 10.1038/s41467-025-66915-1 (PMC12675595; doi:10.1038/s41467-025-66915-1)
Supplement: Supplementary file 1 — Supplementary Information [file 41467_2025_66915_MOESM1_ESM.pdf]

Supplementary Information for "TAPB: An Interventional Debiasing Framework for Alleviating Target Prior Bias in Drug-Target Interaction Prediction"

Gaoming Lin†, Xin Zhang†, Zhonghao Ren, Quan Zou, Prayag Tiwari*, Changjun Zhou*, Yijie Ding*

*Corresponding author(s). E-mail(s): prayag.tiwari@hh.se; zhouchangjun@zjnu.edu.cn; wuxi_dyj@csj.uestc.edu.cn;
†These authors contributed equally to this work.

Supplementary Note 1 and Supplementary Table 1: Statistics of datasets

We summarize the key statistics of the public datasets used in this study in Supplementary Table 1, including the number of Drugs and Targets, the sequence lengths of Drugs and Targets (along with the maximum sequence length, which may consume excessive GPU memory when excessively long), and the total counts as well as the ratios of positive and negative samples for each dataset.

Supplementary Table 1 Statistics of in-domain and cross-domain splits of the BindingDB and BioSNAP datasets, in-domain split of the Davis dataset, and cold split of the Human dataset. Length data is represented as (average length/maximum length). For positive and negative pairs, the values denote (number of pairs/percentage of total pairs)

Dataset	Drug	Target	Drug length	Target length	Pos pairs	Neg pairs
BindingDB in-domain	14643	2623	58.58/680	691.93/7073	20674/0.42	28525/0.58
BioSNAP in-domain	4505	2181	56.71/748	549.95/5183	13830/0.50	13634/0.5
BindingDB cross-domain	8969	1587	57.57/660	692.20/4128	6466/0.39	10241/0.61
BioSNAP cross-domain	3219	1751	52.53/409	542.04/5179	5456/0.51	5217/0.49
Davis in-domain	68	379	63.26/92	801.44/2549	1506/0.14	9597/0.86
Human cold-split	1813	1503	47.32/420	639.74/4655	1995/0.51	1924/0.49

Supplementary Note 2, Supplementary Figure 1 and Supplementary Table 2: Hyperparameter selections

Below, we explain the reasoning behind the selection of key hyperparameters as shown in Supplementary Table 13:

BATCH_SIZE=60: Chosen as the maximum size feasible on a single GPU to optimize memory utilization during training.

MAX_EPOCH=100: Adopted directly from DrugBAN's pretraining protocol [1] to ensure a fair comparison with their work.

LR=2e-4 & WEIGHT_DECAY=1e-4: Selected based on standard transformer optimization practices, e.g. BERT [2], for stable training dynamics.

TARGET_RANDOM_DROP_RATIO=0.7 & MUTATION_RATE=0.2: Determined via ablation studies; values of 70% drop and 20% mutation yielded the best model robustness.

MASK_PROBABILITY=0.15: Follows the established masking strategy used in BERT [2] pretraining.

DICT_SIZE=8: Tuned to achieve an optimal balance between memory requirements and model performance.

DrugEncoder.d_model=256: Increased from 128 based on validation results showing improved molecular feature extraction capability.

DrugEncoder.n_layer=3, DrugEncoder.n_head=8, TransformerDecoder.n_layer=3, TransformerDecoder.n_head=8: Align with typical configurations for base Transformer model architectures.

Activation gelu, DrugEncoder.dropout=0.1, TransformerDecoder.dropout=0.1: Utilize common settings and conventions from Transformer [3].

DrugEncoder.vocab_size=2362: Fixed by the inherent size of the drug token dictionary used in Molformer [4].

PrEncoder.d_model=1280: Matches the output dimension of the pretrained ESM-2 [5] embeddings to enable direct transfer learning.

TransformerDecoder.d_model=256: Ensures dimensionality compatibility with the outputs from the DrugEncoder.

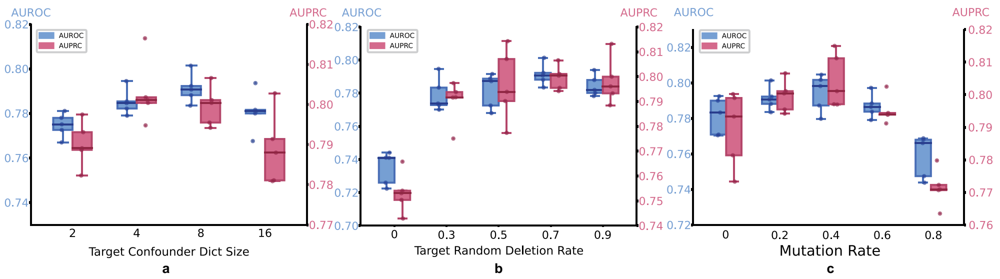

Supplementary Figure 1 Hyperparameter tuning results. (**a**) AUROC and AURPC of TAPB on the cross-domain split of the BioSNAP dataset under various target confounder dictionary sizes. (**b**) AUROC and AURPC of TAPB on the cross-domain split of the BioSNAP dataset under various target random drop ratios. (**c**) AUROC and AURPC of TAPB on the cross-domain split of the BioSNAP dataset under various mutation rates. Experiments were performed with five different random seeds across all datasets. Source data are provided as a Source Data File.

We report the hyperparameter optimization results on the cross-domain split of the BioSNAP dataset for our three key parameters: Target Confounder Dict Size, Target Random Drop Ratio, and Mutation Rate. The results, which include the mean values and standard deviations from five independent runs per configuration with different random seeds, are presented in Supplementary Table 2.

Target confounder dictionary size: The target confounder dictionary plays a critical role in computing $P(Y|\mathbf{D}, do(\mathbf{T}))$ via backdoor adjustment. We evaluated the appropriate dictionary size by comparing TAPB performance on the cross-domain split of the BioSNAP dataset across varying sizes. As shown in Supplementary Figure 1a, TAPB achieved optimal performance with a dictionary size of 8, yielding an average AUROC of 0.79 and AUPRC of 0.80. This configuration demonstrated superior AUPRC compared to other sizes and was consequently selected as the default setting.

Target random deletion ratio: This hyperparameter governs amino acid randomization by deleting features at fixed proportions. We evaluated TAPB performance on the cross-domain split of the BioSNAP dataset across drop ratios ranging from 0 to 0.9. Supplementary Figure 1b shows that performance was lowest without random drop (ratio=0). Introducing any drop ratio (>0) increased AUROC by approximately 4% across configurations. Target random deletion prevents spurious correlation memorization, suppresses target prior bias, and reduces computational costs by shortening

Supplementary Table 2 Detailed hyperparameter optimization results for Target Confounder Dict Size, Target Random Drop Ratio, and Mutation Rate. For clarity, the highest results are indicated in bold.

Value	AUROC	AUPRC
	Target Confounder Dict Size	
2	0.775±0.005	0.790±0.006
4	0.785±0.006	**0.803±0.008**
8	**0.791±0.007**	0.800±0.005
16	0.780±0.009	0.789±0.009
	Target Random Drop Ratio	
0	0.735±0.010	0.753±0.008
0.3	0.779±0.010	0.790±0.009
0.5	0.782±0.011	0.797±0.015
0.7	**0.791±0.007**	**0.800±0.005**
0.9	0.786±0.006	0.798±0.009
	Mutation Rate	
0	0.781±0.010	0.789±0.011
0.2	0.791±0.007	0.800±0.005
0.4	**0.794±0.010**	**0.804±0.008**
0.6	0.787±0.007	0.795±0.004
0.8	0.759±0.012	0.771±0.006

target feature length. We selected a ratio of 0.7 as the default due to its stable predictive performance.

Mutation rate: This hyperparameter governs amino acid mutations during randomization. We evaluated TAPB performance on the cross-domain split of the BioSNAP dataset across mutation rates ranging from 0 to 0.8, with results presented in Supplementary Figure 1c. Model performance demonstrates a unimodal trend: it initially improves, then declines as the mutation rate increases. Peak performance occurs at a mutation rate of 0.4, achieving 0.79 AUROC and 0.80 AUPRC. This indicates that higher mutation rates introduce excessive noise that destabilizes training. We selected a mutation rate of 0.2 as default since it maintains competitive metrics approaching those at 0.4 while exhibiting the lowest standard deviation across runs.

Supplementary Note 3, Supplementary Figure 2: ESM-2 features do not drive DTI prediction bias

In addition to proving that "prior tendency" is the cause of biased predictions in DTI sequence datasets, we also tested whether the stronger features extracted by ESM-2 could lead to biased predictions. For this purpose, we conducted bias tests $(\mathbf{T}, \mathbf{R})$ and $(\mathbf{D}, \mathbf{R})$ on the previously constructed unbiased dataset using the TAPB-Base. As shown in Supplementary Figures 2a and b, TAPB does not exhibit "target bias" when receiving random inputs $\mathbf{R}$, unlike in the BindingDB and BioSNAP datasets. This suggests that in DTI sequence datasets, labels and other priors have a more significant impact on DTI predictions, making them more susceptible to biased predictions.

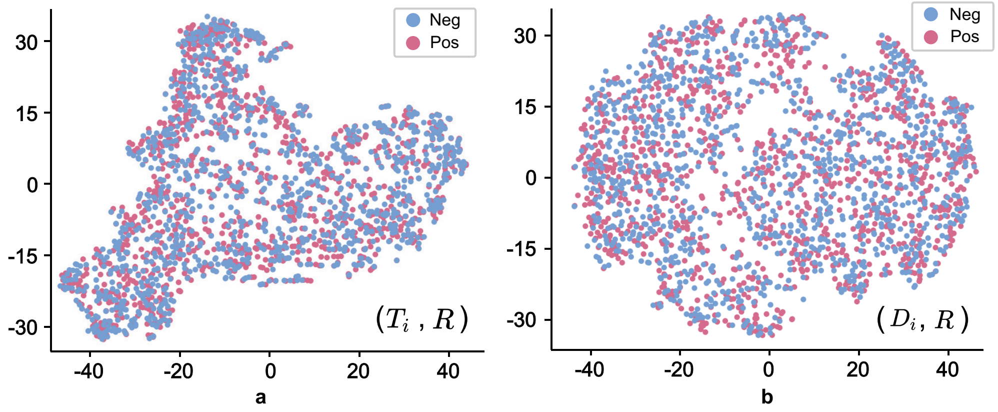

Supplementary Figure 2 T-SNE Visualization of bias tests. (**a**) TAPB "target bias" test on a prior-balanced dataset. (**b**) TAPB "drug bias" test on a prior-balanced dataset.

Supplementary Tables 3-8: Detailed Results

Supplementary Table 3 Comparison of performance on the in-domain splits of BioSNAP, BindingDB, Davis, and the cold split of Human. We report the averaged Youden Index optimized threshold t for every model and the mean and standard deviation of the results for five different seeds. Best results highlighted in bold.

Method	AUROC	AUPRC	Accuracy	Sensitivity	Specificity
		BioSNAP			
TransformerCPI [6](t=0.873)	0.891±0.004	0.896±0.005	0.822±0.005	0.809±0.020	0.835±0.021
MolTrans [7](t=0.687)	0.893±0.008	0.900±0.010	0.819±0.011	0.798±0.020	0.839±0.016
DrugBAN [1](t=0.754)	0.903±0.003	0.907±0.004	0.841±0.001	0.81±0.020	0.868±0.022
PSICHIC [8](t=0.885)	0.92±0.003	0.922±0.004	0.854±0.004	0.840±0.025	0.868±0.024
MlanDTI [9](t=0.584)	0.901±0.02	0.908±0.01	0.829±0.006	0.816±0.014	0.843±0.017
TAPB(t=0.754)	**0.943±0.003**	**0.944±0.003**	**0.881±0.003**	**0.874±0.008**	**0.887±0.011**
		BindingDB			
TransformerCPI [6](t=0.403)	0.948±0.001	0.932±0.002	0.891±0.004	0.881±0.009	0.900±0.005
MolTrans [7](t=0.274)	0.938±0.003	0.913±0.003	0.868±0.008	0.877±0.015	0.862±0.016
DrugBAN [1](t=0.419)	**0.963±0.001**	**0.953±0.001**	**0.908±0.002**	**0.906±0.005**	**0.909±0.005**
PSICHIC [8](t=0.326)	0.948±0.003	0.929±0.007	0.891±0.007	0.890±0.011	0.892±0.001
MlanDTI [9](t=0.455)	0.941±0.005	0.923±0.006	0.868±0.011	0.871±0.005	0.866±0.021
TAPB(t=0.403)	0.961±0.001	0.950±0.002	0.901±0.003	0.899±0.015	0.902±0.014
		Davis			
TransformerCPI [6](t=0.219)	0.842±0.023	0.202±0.041	0.738±0.037	0.836±0.014	0.732±0.039
MolTrans [7](t=0.396)	0.900±0.008	0.341±0.029	0.802±0.038	0.865±0.039	0.798±0.042
DrugBAN [1](t=0.637)	0.886±0.004	0.319±0.025	0.809±0.037	0.831±0.043	0.808±0.041
PSICHIC [8](t=0.523)	0.912±0.005	0.366±0.023	0.848±0.023	0.862±0.029	0.848±0.026
MlanDTI [9](t=0.597)	0.871±0.004	0.304±0.009	0.798±0.017	0.814±0.013	0.798±0.018
TAPB(t=0.521)	**0.933±0.006**	**0.44±0.013**	**0.860±0.014**	**0.871±0.009**	**0.860±0.015**
		Human			
TransformerCPI [6](t=0.403)	0.831±0.025	0.780±0.027	0.768±0.02	0.77±0.081	0.766±0.046
MolTrans [7](t=0.274)	0.821±0.011	0.778±0.010	0.773±0.009	0.727±0.039	0.802±0.034
DrugBAN [1](t=0.419)	0.828±0.018	0.756±0.026	0.750±0.035	0.802±0.068	0.718±0.098
PSICHIC [8](t=0.326)	**0.887±0.013**	**0.826±0.021**	**0.891±0.007**	**0.812±0.044**	**0.836±0.046**
MlanDTI [9](t=0.455)	0.869±0.008	0.805±0.012	0.817±0.016	0.785±0.053	0.837±0.057
TAPB(t=0.403)	0.848±0.021	0.784±0.028	0.767±0.015	0.815±0.044	0.737±0.045

Supplementary Table 4 Comparison of cross-domain performance on BioSNAP, BindingDB datasets. We report the averaged Youden Index optimized threshold t for every model and the mean and standard deviation of the results for five different seeds. Best results highlighted in bold.

Method	AUROC	AUPRC	Accuracy	Sensitivity	Specificity
		BioSNAP			
TransformerCPI [6](t=0.07)	0.603±0.013	0.595±0.011	0.586±0.007	0.652±0.174	0.519±0.186
MolTrans [7](t=0.297)	0.631±0.01	0.632±0.016	0.604±0.013	0.56±0.141	0.649±0.13
DrugBAN+CDAN [1] (t=0.455)	0.685±0.017	0.722±0.010	0.654±0.009	0.523±0.070	0.786±0.040
PSICHIC [8](t=0.262)	0.778±0.016	0.786±0.014	0.724±0.02	0.695±0.046	0.754±0.043
MlanDTI [9](t=0.601)	0.741±0.009	0.775±0.009	0.69±0.011	0.571±0.038	**0.809±0.045**
TAPB(t=0.318)	**0.791±0.007**	**0.800±0.005**	**0.732±0.012**	**0.676±0.075**	0.789±0.083
		BindingDB			
TransformerCPI[6](t=0.158)	0.514±0.029	0.478±0.020	0.517±0.028	0.692±0.248	0.365±0.260
MolTrans[7](t=0.263)	0.605±0.038	0.554±0.044	0.582±0.026	0.638±0.185	0.534±0.179
DrugBAN+CDAN[1](t=0.410)	0.601±0.046	0.57±0.051	0.579±0.039	0.541±0.159	0.612±0.200
PSICHIC [8](t=0.202)	0.619±0.039	0.58±0.033	0.588±0.035	0.779±0.165	0.421±0.196
MlanDTI [9](t=0.326)	0.678±0.035	**0.63±0.047**	0.627±0.025	**0.828±0.082**	0.452±0.108
TAPB(t=0.440)	**0.676±0.016**	0.628±0.029	**0.63±0.016**	0.705±0.155	**0.565±0.158**

Supplementary Table 5 Ablation results on Davis dataset across random seeds.

Version	Vaule1	Vaule2	Value3	Value4	Value5
		AUROC			
TAPB-CNN	0.8996	0.8944	0.8969	0.8907	0.8978
TAPB-Base	0.9107	0.9089	0.9042	0.9161	0.9176
TAPB-R	0.9203	0.9232	0.9218	0.9141	0.9288
TAPB-RM	0.9262	0.9248	0.9217	0.9228	0.9223
TAPB-RM-BA	0.9233	0.9276	0.9238	0.9260	0.9277
TAPB-RM-CAM	0.9261	0.9252	0.9313	0.9269	0.9265
TAPB-Full	0.9383	0.9242	0.9358	0.9373	0.9309
		AUPRC			
TAPB-CNN	0.3505	0.3291	0.3100	0.2959	0.3563
TAPB-Base	0.3557	0.3593	0.3010	0.3593	0.3502
TAPB-R	0.3544	0.3792	0.4308	0.3641	0.4359
TAPB-RM	0.4391	0.4362	0.4086	0.4125	0.4067
TAPB-RM-BA	0.3774	0.3946	0.4437	0.4107	0.4246
TAPB-RM-CAM	0.4435	0.4433	0.4280	0.4363	0.4551
TAPB-Full	0.4510	0.4219	0.4354	0.4544	0.4375

Supplementary Table 6 Ablation results on the cross-domain split of the BioSNAP dataset across random seeds

Version	Vaule1	Vaule2	Value3	Value4	Value5
			AUROC		
TAPB-CNN	0.6481	0.6475	0.6344	0.6620	0.6616
TAPB-Base	0.7536	0.7439	0.7517	0.7396	0.7514
TAPB-R	0.7800	0.7816	0.7936	0.7676	0.7810
TAPB-RM	0.7805	0.7962	0.7741	0.7773	0.7807
TAPB-RM-BA	0.7815	0.7688	0.7813	0.7895	0.8008
TAPB-RM-CAM	0.7974	0.7936	0.7808	0.7934	0.7882
TAPB-Full	0.7907	0.7835	0.7923	0.8014	0.7882
			AUPRC		
TAPB-CNN	0.6481	0.6415	0.6131	0.6484	0.6651
TAPB-Base	0.7622	0.7581	0.7607	0.7485	0.7710
TAPB-R	0.7809	0.7914	0.8028	0.7811	0.7880
TAPB-RM	0.7949	0.8035	0.7826	0.7897	0.7838
TAPB-RM-BA	0.7993	0.7860	0.7931	0.7903	0.8071
TAPB-RM-CAM	0.8022	0.7947	0.7908	0.8107	0.7942
TAPB-Full	0.8011	0.7942	0.7955	0.8066	0.8004

Supplementary Table 7 Ablation results on the cross-domain split of the BindingDB dataset across random seeds

Version	Vaule1	Vaule2	Value3	Value4	Value5
			AUROC		
TAPB-CNN	0.6372	0.6092	0.6585	0.5454	0.6595
TAPB-Base	0.6714	0.6572	0.6513	0.6480	0.6549
TAPB-R	0.6857	0.6584	0.6587	0.6763	0.6664
TAPB-RM	0.6789	0.6611	0.6550	0.6832	0.6784
TAPB-RM-BA	0.6598	0.6620	0.6909	0.6688	0.6827
TAPB-RM-CAM	0.6625	0.6667	0.6746	0.6651	0.6706
TAPB-Full	0.6853	0.6577	0.6641	0.6986	0.6731
			AUPRC		
TAPB-CNN	0.5472	0.5856	0.6101	0.4905	0.6087
TAPB-Base	0.6119	0.6027	0.5578	0.5873	0.5898
TAPB-R	0.6301	0.6009	0.6176	0.6155	0.5922
TAPB-RM	0.6124	0.5973	0.5950	0.6457	0.6414
TAPB-RM-BA	0.6106	0.6101	0.6375	0.5949	0.6123
TAPB-RM-CAM	0.5837	0.6042	0.6032	0.6016	0.6093
TAPB-Full	0.6376	0.5937	0.6298	0.6701	0.6074

Supplementary Table 8 Ablation results on the cross-domain split of the BindingDB dataset across random seeds

Version	Vaule1	Vaule2	Value3	Value4	Value5
		AUROC			
TransformerCPI	0.5906	0.6071	0.5963	0.6229	0.5959
TransformerCPI w/ random deletion	0.6846	0.6981	0.6878	0.6926	0.6832
DrugBAN	0.5767	0.6167	0.6246	0.6245	0.5940
DrugBAN w/ random deletion	0.6191	0.5949	0.6318	0.6222	0.6379
		AUPRC			
TransformerCPI	0.5862	0.5936	0.5969	0.6132	0.5869
TransformerCPI w/ random deletion	0.6960	0.7103	0.7115	0.7033	0.6987
DrugBAN	0.5721	0.6306	0.6216	0.6321	0.6017
TransformerCPI w/ random deletion	0.6041	0.5964	0.6234	0.6128	0.6363

Supplementary Note 4, Supplementary Tables 9-16: Experiment settings

We provide the default hyperparameters used in our experiments on both in-domain and cross-domain splits of BioSNAP, BindingDB, in-domain split of Davis, cold split of Human, and two counter-prior training sets.

Supplementary Table 9 Default hyperparameter settings for TransformerCPI.

Model	Hyperparameter	Value
TransformerCPI	batch	64
	lr	1e-4
	weight_decay	1e-4
	lr_decay	1.0
	iteration	100
	protein_dim	100
	atom_dim	34
	hid_dim	64
	n_layers	3
	n_heads	8
	pf_dim	256
	dropout	0.1
	kernel_size	7

Supplementary Table 10 Hyperparameter settings for MolTrans.

Model	Hyperparameter	Value
MolTrans	batch_size	16
	input_dim_drug	23532
	input_dim_target	16693
	train_epoch	13
	max_drug_seq	50
	max_protein_seq	545
	emb_size	384
	dropout_rate	0.1
	scale_down_ratio	0.25
	growth_rate	20
	transition_rate	0.5
	num_dense_blocks	4
	kernal_dense_size	3
	intermediate_size	1536
	num_attention_heads	12
	attention_probs_dropout_prob	0.1
	hidden_dropout_prob	0.1
	flat_dim	78192

Supplementary Table 11 Hyperparameter settings for DrugBAN on in-domain splits of BioSNAP, BindingDB, and Davis, cold split of Human, and two counter-prior training sets.

Model	Hyperparameter	Value
DrugBAN	BATCH_SIZE	32
	MAX_EPOCH	100
	LR	5e-5
	NODE_IN_FEATS	75
	DRUG.HIDDEN_LAYERS	[128, 128, 128]
	DRUG.NODE_IN_EMBEDDING	128
	DRUG.MAX_NODES	290
	PROTEIN.NUM_FILTERS	[128, 128, 128]
	PROTEIN.KERNEL_SIZE	[3, 6, 9]
	PROTEIN.EMBEDDING_DIM	128
	PROTEIN.PADDING	True
	BCN.HEADS	2
	DECODER.IN_DIM	256
	DECODER.HIDDEN_DIM	512
	DECODER.OUT_DIM	128
	DECODER.BINARY	1

Supplementary Table 12 Default hyperparameter settings for DrugBAN+CDAN on cross-domain datasets.

Model	Hyperparameter	Value
DrugBAN+CDAN	BATCH_SIZE	32
	MAX_EPOCH	100
	LR	1e-4
	DA_LR	5e-5
	RANDOM_DIM	256
	INIT_EPOCH	10
	NODE_IN_FEATS	75
	DRUG.HIDDEN_LAYERS	[128, 128, 128]
	DRUG.NODE_IN_EMBEDDING	128
	DRUG.MAX_NODES	290
	PROTEIN.NUM_FILTERS	[128, 128, 128]
	PROTEIN.KERNEL_SIZE	[3, 6, 9]
	PROTEIN.EMBEDDING_DIM	128
	PROTEIN.PADDING	True
	BCN.HEADS	2
	DECODER.IN_DIM	256
	DECODER.HIDDEN_DIM	512
	DECODER.OUT_DIM	128
	DECODER.BINARY	2

Supplementary Table 13 Default hyperparameter settings for TAPB.

Model	Hyperparameter	Value
TAPB	BATCH_SIZE	60
	MAX_EPOCH	100
	LR	2e-4
	WEIGHT_DECAY	1e-4
	TARGET_RANDOM_DROP_RATIO	0.7
	MUTATION_RATE	0.2
	MASK_PROBABILITY	0.15
	DICT_SIZE	8
	DrugEncoder.d_model	256
	DrugEncoder.n_layer	3
	DrugEncoder.n_head	8
	activation	gelu
	DrugEncoder.dropout	0.1
	DrugEncoder.vocab_size	2362
	PrEncoder.d_model	1280
	TransformerDeocder.d_model	128
	TransformerDeocder.n_layer	3
	TransformerDeocder.n_head	8
	TransformerDeocder.activation	gelu
	TransformerDeocder.dropout	0.1

Supplementary Table 14 Hyperparameter settings for PSICHIC.

Model	Hyperparameter	Value
PSICHIC	batch_size	10
	epochs	100
	lrate	1e-4
	weight_decay	1e-4
	clip	1
	betas	$[0.9, 0.999]$
	eps	1e-08
	min_lrate	0
	warmup_iters	0
	lr_decay_iters	290
	mol_in_channels	43
	prot_in_channels	33
	pror_evo_channels	1280
	hidden_channes	200
	pre_layers	2
	post_layers	1
	total_layer	3
	dropout	0
	dropout_attn_score	0.2
	heads	5

Supplementary Table 15 Hyperparameter settings for MlanDTI.

Model	Hyperparameter	Value
MLanDTI	batch	64
	epoch	40
	protein_dim	1024
	hid_dim	128
	atom_dim	32
	n_heads	8
	n_enlayers	2
	n_delayers	2
	dropout	0.2
	settings_cls.protein_dim	1024
	settings_cls.atom_dim	32
	settings_cls.hid_dim	128
	settings_cls.dropout	0.2
	lr	0.001
	weight_decay	0.0001
	decay_interval	5
	lr_decay	0.5

Supplementary Table 16 Hyperparameter settings for DrugBAN_Non_DA.

Model	Hyperparameter	Value
DrugBAN_Non_DA	BATCH_SIZE	32
	MAX_EPOCH	100
	LR	5e-5
	NODE_IN_FEATS	75
	DRUG.HIDDEN_LAYERS	[128, 128, 128]
	DRUG.NODE_IN_EMBEDDING	128
	DRUG.MAX_NODES	290
	PROTEIN.NUM_FILTERS	[128, 128, 128]
	PROTEIN.KERNEL_SIZE	[3, 6, 9]
	PROTEIN.EMBEDDING_DIM	128
	PROTEIN.PADDING	True
	BCN.HEADS	2
	DECODER.IN_DIM	256
	DECODER.HIDDEN_DIM	512
	DECODER.OUT_DIM	128
	DECODER.BINARY	1

Supplementary Note 5, Supplementary Figure 3: Extend data statistics

We quantified the ”prior tendency” and the overall ”prior tendency” in the in-domain split of Davis and the cold split of Human. As illustrated in Supplementary Figures 3a, b and c, the Davis dataset exhibits a certain ”target prior bias”, while a more pronounced ”drug prior bias” is observed in the Human dataset. Statistical analysis confirmed significant deviations for both biases ($P_t = 0.000$, $P_d = 0.000$).

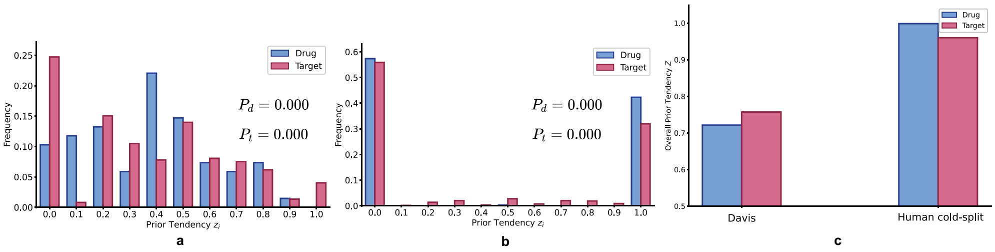

Supplementary Figure 3 Statistics of Davis and Human datasets. (**a**) ”Prior tendency“ frequency distributions of z_i for drugs and targets in the Davis training set. (**b**) ”Prior tendency“ frequency distribution of z_i for drugs and targets in the Human training set. (**c**) Overall ”prior tendency” for labels associated with drugs and targets across Davis and Human datasets. P-values were derived from a one-sided permutation test with 1000 iterations (P_d for drugs, P_t for targets), with no adjustments for multiple comparisons. Source data are provided as a Source Data File.

Supplementary Algorithm 1: TAPB Pseudocode

Supplementary Algorithm 1 TAPB Forward Propagation

Require:

Input drugs: input_drugs = {input_ids, attention_mask}
Input proteins: input_proteins
Protein mask: pr_mask
Masked drugs: masked_drugs
c_i ← K-means clustering of ESM2 features ▷ Precomputed
p_ci : The proportion of each cluster in K-means as a percentage of the total

Ensure:

Output: {logits, fusion_f, attn_map, drug_mlm_logits}
Step 1: Encode Target
freqs_cis ← precompute_freqs_cis($d\_model//n\_heads$, 4000)
drug_f ← drug_encoder(input_drugs, freqs_cis)
▷ Generates drug features via Transformer encoder
Step 2: Encode Protein
pr_f ← input_proteins
pr_f ← confounder_alignment(pr_f, confounder$z$)
Step 3: Fusion
fusion_f, attn_map ← aggregator(drug_f, pr_f)
▷ Fuses features via Transformer decoder
Step 4: MLM
drug_f_mlm ← drug_encoder(masked_drugs, freks_cis)
drug_mlm_logits ← MLMHead(drug_f_mlm)
Step 5: Classification
c_i ← reshape_mean(fusion_f) ▷ Group by confounders
logits ← classifier(c_i) ▷ Predict $P(Y|D, T, c_i)$
logits ← backdoor_adjustment(logits, p_ci) ▷ Compute $P(Y|D, do(T))$
Return {logits, fusion_f, attn_map, drug_mlm_logits}

Supplementary Note 6, Supplementary Tables 17-18: Comparison with UdanDTI

The focus of our method is to achieve exceptional cross-domain generalization capability using only the source domain training set. Supplementary Table 17 demonstrates a fair comparison, showing that TAPB exhibits comparable performance to UdanDTI, with a slight advantage on the BioSNAP in-domain split. Notably, the zero-shot performance of TAPB surpasses that of UdanDTI on cross-domain splits without requiring UDA techniques. Specifically, for cross-domain splits, TAPB exceeds UdanDTI by 3.5% in AUROC and 2.7% in AUPRC on BioSNAP, and by 3.5% in AUROC and 5.7% in AUPRC on BindingDB, demonstrating that TAPB inherently possesses generalization ability.

We adapted and enhanced the core data-centric idea of UdanDTI by fine-tuning the TAPB_S model on fewer target domain data. As shown in Supplementary Table

Supplementary Table 17 Comparison of TAPB and UdanDTI performance on BioSNAP, BindingDB datasets. We directly cite the mean and standard deviation of UdanDTI from the UdanDTI paper [10], with the best results highlighted in bold.

Dataset	Method	AUROC	AUPRC
BioSNAP in-domain split	UdanDTI	0.941±0.003	0.942±0.004
	TAPB	**0.943±0.003**	**0.944±0.003**
BindingDB in-domain split	UdanDTI	**0.965±0.001**	**0.955±0.001**
	TAPB	0.961±0.001	0.950±0.002
BioSNAP cross-domain split	UdanDTI	0.756±0.013	0.773±0.021
	TAPB	**0.791±0.007**	**0.800±0.005**
BindingDB cross-domain split	UdanDTI	0.636±0.021	0.571±0.027
	TAPB	**0.676±0.016**	**0.628±0.029**

18, our model achieved a significant performance improvement: on the BioSNAP cross-domain split, TAPB_S increased AUROC and AUPRC by 2.1% and 2.5%, respectively; on BindingDB, the corresponding gains were 16.4% in AUROC and 18.3% in AUPRC.

Supplementary Table 18 Comparison of TAPB_S and UdanDTI_MCD performance on BioSNAP and BindingDB datasets. We directly cite the mean and standard deviation of UdanDTI_MCD from the UdanDTI paper [10], with the best results highlighted in bold.

Dataset	Method	AUROC	AUPRC
BioSNAP cross-domain split	UdanDTI_MCD	0.805±0.011	**0.825±0.008**
	TAPB_S	**0.812±0.009**	**0.825±0.015**
BindingDB cross-domain split	UdanDTI_MCD	0.713±0.017	0.671±0.019
	TAPB_S	**0.840±0.014**	**0.811±0.017**

Supplementary References

[1] Bai, P., Miljković, F., John, B., Lu, H.: Interpretable bilinear attention network with domain adaptation improves drug–target prediction. Nature Machine Intelligence **5**(2), 126–136 (2023)

[2] Kenton, J.D.M.-W.C., Toutanova, L.K.: Bert: Pre-training of deep bidirectional transformers for language understanding. In: Proceedings of naacL-HLT, vol. 1, p. 2 (2019). Minneapolis, Minnesota

[3] Vaswani, A., Shazeer, N., Parmar, N., Uszkoreit, J., Jones, L., Gomez, A.N., Kaiser, Ł., Polosukhin, I.: Attention is all you need. Advances in neural information processing systems **30** (2017)

[4] Ross, J., Belgodere, B., Chenthamarakshan, V., Padhi, I., Mroueh, Y., Das, P.: Large-scale chemical language representations capture molecular structure and properties. Nature Machine Intelligence **4**(12), 1256–1264 (2022) https://doi.org/10.1038/s42256-022-00580-7

[5] Lin, Z., Akin, H., Rao, R., Hie, B., Zhu, Z., Lu, W., Smetanin, N., Santos Costa, A., Fazel-Zarandi, M., Sercu, T., Candido, S., et al.: Language models of protein sequences at the scale of evolution enable accurate structure prediction. bioRxiv (2022)

[6] Chen, L., Tan, X., Wang, D., Zhong, F., Liu, X., Yang, T., Luo, X., Chen, K., Jiang, H., Zheng, M.: Transformercpi: improving compound–protein interaction prediction by sequence-based deep learning with self-attention mechanism and label reversal experiments. Bioinformatics **36**(16), 4406–4414 (2020)

[7] Huang, K., Xiao, C., Glass, L.M., Sun, J.: Moltrans: molecular interaction transformer for drug–target interaction prediction. Bioinformatics **37**(6), 830–836 (2021)

[8] Koh, H.Y., Nguyen, A.T., Pan, S., May, L.T., Webb, G.I.: Physicochemical graph neural network for learning protein–ligand interaction fingerprints from sequence data. Nature Machine Intelligence **6**(6), 673–687 (2024)

[9] Xie, Z., Tu, S., Xu, L.: Multilevel attention network with semi-supervised domain adaptation for drug-target prediction. In: Proceedings of the AAAI Conference on Artificial Intelligence, vol. 38, pp. 329–337 (2024)

[10] Zhang, P., Ma, J., Chen, T.: Escaping the drug-bias trap: Using debiasing design to improve interpretability and generalization of drug-target interaction prediction. IEEE Transactions on Computational Biology and Bioinformatics **22**(4), 1902–1911 (2025) https://doi.org/10.1109/TCBBIO.2025.3576488
