# Supplementary material for: TAPB: an interventional debiasing framework for alleviating target prior bias in drug-target interaction prediction
Source: Nat Commun. 2025 Dec 2;16:10867. doi: 10.1038/s41467-025-66915-1 (PMC12675595; doi:10.1038/s41467-025-66915-1)
Supplement: Supplementary file 2 — Reporting Summary [file 41467_2025_66915_MOESM2_ESM.pdf]

nature portfolio

Corresponding author(s): Prayag Tiwari, Changjun Zhou, Yijie Ding

Last updated by author(s): Oct 20, 2025

Reporting Summary

Nature Portfolio wishes to improve the reproducibility of the work that we publish. This form provides structure for consistency and transparency in reporting. For further information on Nature Portfolio policies, see our Editorial Policies and the Editorial Policy Checklist.

Statistics

For all statistical analyses, confirm that the following items are present in the figure legend, table legend, main text, or Methods section.

n/a	Confirmed	
☐	☒	The exact sample size (*n*) for each experimental group/condition, given as a discrete number and unit of measurement
☐	☒	A statement on whether measurements were taken from distinct samples or whether the same sample was measured repeatedly
☒	☐	The statistical test(s) used AND whether they are one- or two-sided<br>*Only common tests should be described solely by name; describe more complex techniques in the Methods section.*
☒	☐	A description of all covariates tested
☒	☐	A description of any assumptions or corrections, such as tests of normality and adjustment for multiple comparisons
☐	☒	A full description of the statistical parameters including central tendency (e.g. means) or other basic estimates (e.g. regression coefficient) AND variation (e.g. standard deviation) or associated estimates of uncertainty (e.g. confidence intervals)
☐	☒	For null hypothesis testing, the test statistic (e.g. *F*, *t*, *r*) with confidence intervals, effect sizes, degrees of freedom and *P* value noted<br>*Give P values as exact values whenever suitable.*
☒	☐	For Bayesian analysis, information on the choice of priors and Markov chain Monte Carlo settings
☒	☐	For hierarchical and complex designs, identification of the appropriate level for tests and full reporting of outcomes
☒	☐	Estimates of effect sizes (e.g. Cohen's *d*, Pearson's *r*), indicating how they were calculated

Our web collection on statistics for biologists contains articles on many of the points above.

Software and code

Policy information about availability of computer code

Data collection	We didn't use any commecial softwares for data collection. All used datasets are available at https://github.com/GaomingL1n/TAPB
Data analysis	We use the following packages for statistics and visualization: Python 3.9, PyTorch 2.2.1 Numpy 1.23.0, Scikit-learn 1.2.1, Pandas 2.2.3, RDkit 2024.3.3, Matplotlib 3.5.0. All visualization details, custom codes for analysis details are available at our GitHub repository (https://github.com/GaomingL1n/TAPB). Docking calculations were performed with AutoDock Vina (v 1.2.5). 2D ligand-protein interaction diagrams were generated using the Ligand Interaction Diagram module in Maestro (v13.5, Schrödinger), and 3D ligand-protein interaction diagrams were prepared in PyMol(v2.5).

For manuscripts utilizing custom algorithms or software that are central to the research but not yet described in published literature, software must be made available to editors and reviewers. We strongly encourage code deposition in a community repository (e.g. GitHub). See the Nature Portfolio guidelines for submitting code & software for further information.

Data

Policy information about availability of data

All manuscripts must include a data availability statement. This statement should provide the following information, where applicable:

- Accession codes, unique identifiers, or web links for publicly available datasets
- A description of any restrictions on data availability
- For clinical datasets or third party data, please ensure that the statement adheres to our policy

The BioSNAP, BindingDB, and Human datasets are publicly available at DrugBAN GitHub respository (https://github.com/peizhenbai/DrugBAN). Davis dataset is

available at ConPLex GiHub respository (https://github.com/samsledje/ConPLex_dev). All datasets are also available at our Github respository (https://github.com/GaomingL1n/TAPB).

Research involving human participants, their data, or biological material

Policy information about studies with human participants or human data. See also policy information about sex, gender (identity/presentation), and sexual orientation and race, ethnicity and racism.

Reporting on sex and gender	NA
Reporting on race, ethnicity, or other socially relevant groupings	NA
Population characteristics	NA
Recruitment	NA
Ethics oversight	NA

Note that full information on the approval of the study protocol must also be provided in the manuscript.

Field-specific reporting

Please select the one below that is the best fit for your research. If you are not sure, read the appropriate sections before making your selection.

- [x] Life sciences
- [ ] Behavioural & social sciences
- [ ] Ecological, evolutionary & environmental sciences

For a reference copy of the document with all sections, see nature.com/documents/nr-reporting-summary-flat.pdf

Life sciences study design

All studies must disclose on these points even when the disclosure is negative.

Sample size	The study incorporated six datasets to ensure robust methdological rigor and comparability with existing literature. Specifically, we utilized several well-established publicly available datasets: (a)BioSNAP in-domain (n=27,464), (b)BioSNAP cross-domain (n=10,673), (c)BindingDB in-domain (n=49,199), (d) BindingDB cross-domain (n=16,707), (e) Davis (n=11,107), and (f) Human (n=3,919). These datasets are widely used, with sample sizes optimized through previous works to ensure appropriate statistical power for analysis. To further validate the identified prior tendency, we re-split the BindingDB in-domain training set into two counter-prior subsets: a drug-biased training set(n=2,271) and a balanced training set (n=2,271). The selection of these sample sizes was informed by several considerations: (i) the datasets are based on publicly available resources with established reliability and credibility, (ii) all interaction data used has been experimentally validated, and (iii) the sample sizes are sufficient to rigorously test our hypothesis that prior tendency induces biased predictions. Statistical significance was assessed using a one-sided permutation test (B=1000, to balance statistical percision with computational feasibility), with no adjustments for comparisons. For all comparison experiments, we performed five independent experiments with randomly selected samples.
Data exclusions	No data was excluded in this work.
Replication	The provided codes of our model can reproduce the work results.
Randomization	We allocate samples randomly.
Blinding	We use a computer to randomly allocate.

Reporting for specific materials, systems and methods

We require information from authors about some types of materials, experimental systems and methods used in many studies. Here, indicate whether each material, system or method listed is relevant to your study. If you are not sure if a list item applies to your research, read the appropriate section before selecting a response.

Materials & experimental systems

n/a	Involved in the study	
x		Antibodies
x		Eukaryotic cell lines
x		Palaeontology and archaeology
x		Animals and other organisms
x		Clinical data
x		Dual use research of concern
x		Plants

Methods

n/a	Involved in the study	
x		ChIP-seq
x		Flow cytometry
x		MRI-based neuroimaging

Plants

Seed stocks	NA
Novel plant genotypes	NA
Authentication	NA
